# Ahead of the ambulance: Optimizing volunteer training

**DOI:** 10.1007/s10729-026-09771-9

**Published:** 2026-06-11

**Authors:** Brian Overbeek, Pieter L. van den Berg, Caroline J. Jagtenberg, Rob van der Mei

**Affiliations:** 1 Vrije Universiteit Amsterdam, School of Business and Economics, De Boelelaan 1105, Amsterdam, 1081 HV The Netherlands; 2 Rotterdam School of Management, Erasmus University, Burgemeester Oudlaan 50, Rotterdam, 3062 PA The Netherlands; 3 CWI, Stochastics, Science Park 123, Amsterdam, 1098 XG The Netherlands; 4 Vrije Universiteit Amsterdam, Faculty of Science, De Boelelaan 1105, Amsterdam, 1081 HV The Netherlands

**Keywords:** Health care, Optimization, Emergency medical services, Community first responders, Volunteers, Resource allocation

## Abstract

Emergency response for medical incidents is increasingly extended by community first responder (CFR) systems that dispatch nearby trained volunteers. The implementation of CFR systems has led to significant decreases in emergency response times, especially in rural areas where ambulances take longer to arrive. CFR systems that dispatch volunteers to various emergency types can increase their effectiveness by training their volunteers, enabling these volunteers to provide first aid for more emergency types. We study the problem of optimizing a CFR system’s training strategy to maximize its effectiveness given a limited budget, where the effectiveness is measured by the probability that at least one volunteer arrives before the ambulance for any given incident. We introduce an optimization model that explicitly accounts for the heterogeneous nature of volunteers’ availability and locations, as well as a solution approach that efficiently obtains optimal solutions for realistically-sized instances. We apply the optimization approach to a CFR system operating in Lincolnshire, United Kingdom. The results show that the optimization approach yields substantially larger improvements in the CFR system’s effectiveness compared to several intuitive greedy training strategies. Additionally, dispatch restrictions to limit the workload of volunteers are shown to have important implications for the optimal training strategy.

**Supplementary Information:**

The online version contains supplementary material available at 10.1007/s10729-026-09771-9.

## Highlights

• Community first responder (CFR) systems that dispatch trained volunteers to medical incidents are increasingly implemented to decrease emergency response times. We introduce an optimization approach to optimize the training strategy of a CFR system under a limited budget to maximize its effectiveness.

• We introduce a new metric for the effectiveness of CFR systems that explicitly considers ambulance response times.

• We develop an integer programming-based solution approach that efficiently solves realistically-sized instances and that can also be used for expected coverage optimization.

• Results of a case study on an existing CFR system show that the optimization approach spends training budgets much more efficiently than several intuitive greedy training strategies.

• We characterize the optimal training strategy for this case study and analyze the impact of dispatch restrictions.

**Supplementary Information:**

The online version contains supplementary material available at 10.1007/s10729-026-09771-9.

## Introduction

The probability of surviving severe medical emergencies such as out-of-hospital cardiac arrests (OHCAs) can depend heavily on the emergency response time. Numerous countries decrease these response times by dispatching community first responders (CFRs) in parallel to the ambulance services. CFRs are medically trained volunteers who can be alerted when incidents occur in their vicinity. Available CFRs then go to the incident to provide immediate first aid until an ambulance arrives. Throughout this paper, we use the terms “CFRs” and “volunteers” interchangeably.

Existing CFR systems have shown that volunteers could arrive before the ambulance in a substantial percentage of incidents and significantly reduce response times [1–4]. The effectiveness of CFR systems is believed to be largest in rural areas, where emergency response times are typically longer [5, 6] and survival rates are significantly lower than in urban areas [7]. Recent studies confirmed these beliefs by showing that volunteers arrive before the ambulance most often in rural areas and that the gap between volunteer and ambulance arrival times is largest in these areas [8, 9]. CFR systems thus have the potential to reduce inequalities between urban and rural areas.

Most CFR systems dispatch volunteers only to OHCAs as these are extremely time-sensitive and require only limited training. However, dispatching volunteers could be effective for other emergency types as well. In the UK, for example, many volunteers have been trained to provide medical assistance for traumatic emergencies, neurological emergencies, and emergencies caused by cardiovascular diseases [6]. Volunteers could also be trained to intervene during opioid overdoses and allergic emergencies [10]. Hence, volunteer response is not always restricted to OHCAs.

Naturally, the medical skills a volunteer must have to provide first aid vary across emergency types. However, volunteers’ medical skills can differ considerably, so not all volunteers can provide first aid for all emergencies. Therefore, unlike OHCA-only CFR systems, more general CFR systems typically group volunteers based on their level of medical training such that volunteers with the same training level can provide first aid for the same emergency types.

The training levels of volunteers then directly determine to which emergencies these volunteers can be dispatched. The more volunteers possess the required medical skills to attend a certain incident, the higher the probability that at least one will be available to provide first aid. Therefore, a volunteer base’s skills directly affect the CFR system’s effectiveness. And conversely, one can increase a CFR system’s effectiveness by training its volunteers.

The budget available for training volunteers, however, is typically limited as many CFR systems are operated by charities. How the available budget is spent directly affects the system’s effectiveness. Therefore, this study focuses on the problem of optimizing the training strategy of a CFR system by deciding which volunteers receive which training given a limited budget to maximize the system’s effectiveness.

Two factors complicate the training decisions substantially. First, volunteers’ availability (i.e., whether they are available to respond when alerted) and locations are highly uncertain, cannot be controlled, and can vary substantially among individuals. Whereas many systems within the emergency medical services (EMS) have homogeneous providers of service for which their location and operations can be controlled explicitly, volunteers are inherently heterogeneous and determine themselves when they are available and from where they respond. Secondly, there are often large geographical differences in the number of volunteers, the number of incidents, and ambulance response times, all of which impact the potential benefit of training a volunteer. Finding the optimal training decisions is consequently often nontrivial.

To manage this complexity, we introduce an optimization model to decide what training to provide to which volunteer to maximize a CFR system’s effectiveness with a given budget, accounting for the unique characteristics of volunteers and the above-mentioned geographical features. We introduce a new metric for the effectiveness of CFR systems: the *expected relief*, defined as the probability that *at least* one volunteer arrives before the ambulance. This objective not only reflects that volunteers are particularly valuable when arriving before the ambulance but can also steer decision-making towards reducing inequalities resulting from unevenly distributed ambulance response times. Many EMS systems focus on maximizing efficiency, but this typically has the inadvertent consequence of rural areas being worse off than urban areas [11–13]. Optimizing the expected relief provides an explicit incentive to train volunteers in rural areas because these volunteers are more likely to arrive before the ambulance [8, 9], potentially reducing inequalities between urban and rural areas.

We present a nonlinear integer programming formulation of the optimization problem and introduce a solution approach that efficiently obtains optimal solutions for realistically-sized instances. This solution approach uses a piecewise-linear approximation and requires a commercial optimization solver such as Gurobi [14]. Therefore, our Online supplement presents an alternative solution approach that efficiently finds near-optimal solutions using open-source solvers. The problem formulation and solution approach can also be used to optimize *coverage*, a performance metric commonly used in EMS systems that measures the fraction of incidents reached within a pre-specified response-time target. More specifically, the introduced optimization model is mathematically equivalent to an expected coverage optimization model with server-specific availability. Finally, we demonstrate the performance of our optimization approach through a case study involving the CFR system of the Lincolnshire Integrated Voluntary Emergency Service (LIVES) [15].

Our main contribution is introducing the new and socially relevant optimization problem of deciding how to train volunteers for a CFR system under a limited budget to maximize its effectiveness. Despite the rapidly increasing scientific interest in CFR systems, studies optimizing decision-making within such systems are extremely limited, especially for long-term decision-making. Moreover, we develop an efficient solution approach that can also be used to optimize expected coverage with server-specific availability, something which has been done only for very small instances to date in existing literature. Using a case study on the CFR system of LIVES, we show that the introduced optimization model substantially outperforms several intuitive greedy training strategies.

The remainder of this paper is organized as follows. Section 2 discusses the literature relevant to this study. Section 3 presents the optimization approach, while Section 4 provides an illustrative example demonstrating this approach and the difficulty of finding the optimal training decisions. Sections 5 and 6 discuss the case study and the corresponding results. Finally, Section 7 provides concluding remarks.

## Related work

We first discuss literature on optimizing the effectiveness of CFR systems in Section 2.1. Moreover, we discuss literature related to coverage optimization problems within EMS systems in Section 2.2 as the introduced optimization approach can also be used to maximize expected coverage.

### CFR system optimization

Although the introduction of CFR systems has been associated with considerable improvements in health outcomes - such as survival rates following OHCA [16] - very little effort has been made to optimize the effectiveness of such systems. As observed by van den Berg et al. [17], the vast majority of quantitative studies involving CFR systems analyze the systems’ impact retrospectively and do not apply optimization methods to enhance their effectiveness.

Studies introducing optimization models for CFR systems almost exclusively focus on the dispatch process. Optimizing dispatch decisions is highly complex due to the high degree of uncertainty in volunteers’ availability and behavior, particularly when multiple tasks must be performed, like providing cardiopulmonary resuscitation (CPR) and fetching an automated external defibrillator (AED) for an OHCA. Additionally, as argued by Henderson et al. [18], it is also important to take into account issues such as volunteer fatigue.

Matinrad et al. [19] developed an online greedy algorithm to assign tasks to optimize OHCA patients’ survival probability whilst accounting for uncertainity in volunteer availability and task compliance. An offline algorithm for a similar problem aiming to minimize the weighted sum of task start times was introduced by Matinrad and Andersson Granberg [20]. A different approach to improve dispatch decisions for an OHCA-focused CFR system was adopted by Slaa [21], who developed a simulation model to evaluate various dispatch policies while accounting for different sources of uncertainty.

The dispatch process focuses on short-term decision-making. Contrarily, the problem of optimizing volunteer training focuses on long-term decision-making. Whereas volunteers’ locations are often known with great accuracy in the short term due to volunteers providing their real-time location, there is considerably more uncertainty in their future whereabouts. Resultingly, in general, optimizing short-term and long-term decision-making within CFR systems require different approaches.

To date, only van den Berg et al. [17] focused op optimizing long-term decision-making within CFR systems. They analytically derived the response-time distribution of the first-arriving volunteer by modeling the presence of volunteers as a Poisson point process. This response-time distribution was subsequently incorporated into an optimization approach to obtain the spatial distribution of volunteers maximizing incident coverage or patient survival.

Although van den Berg et al. [17] is highly relevant to this study, their optimization approach cannot be applied to the problem of optimizing CFR training. This is because they consider volunteers homogeneous in their medical skills and availability. When training volunteers, however, it is crucial to consider such differences in volunteer availability and medical skills. Therefore, we introduce a new optimization approach that considers medical skills and availability to be volunteer-specific.

### Coverage optimization models in EMS systems

This section discusses coverage-based optimization models used for EMS systems that are most relevant to this study. General overviews of numerous coverage-based optimization problems are provided by Brotcorne et al. [22], Li et al. [23], Farahani et al. [24], and Bélanger et al. [25].

One of the first and most influential coverage-based optimization models is the Maximum Coverage Location Problem (MCLP) introduced by Church and ReVelle [26]. The MCLP maximizes the number of demand points covered for a fixed number of servers to be located. One major limitation of the MCLP, however, is that servers are assumed to be always available. Probabilistic coverage models in which servers are modeled to be unavailable a certain fraction of the time, the so-called busy fractions, are commonly used to overcome this limitation. The most widely adopted probabilistic model is the Maximum Expected Coverage Location Problem (MEXCLP) introduced by Daskin [27], which locates servers to maximize expected covered demand.

The MEXCLP makes several simplifying assumptions: all servers have the same busy fraction, servers operate independently, and the busy fractions do not depend on the decisions of the optimization problem. These assumptions do not hold in many EMS applications, most notably ambulance location planning [28]. Not addressing these assumptions can result in inefficient resource deployments for these applications, while significant increases in coverage can be obtained when these assumptions are addressed properly [29]. Resultingly, many studies have focused on relaxing the MEXCLP’s simplifying assumptions.

Various studies have incorporated server-specific busy fractions into the MEXCLP [30–33]. However, doing so substantially increases the problem complexity, causing almost all studies to adopt heuristic approaches to find good rather than optimal solutions.

The most common approach to relax the assumption of server independence is to incorporate the hypercube model of Larson [34], or an approximation thereof (e.g. Larson [35], Jarvis [36], Budge [37]), into the MEXCLP. This hypercube model quantifies the effects of server dependency and accurately calculates server-specific busy fractions by modeling a configuration of servers as a spatial queueing system. Studies that have incorporated a hypercube model into variants of the MEXCLP include Batta et al. [28], Saydam and Aytuğ [32], Ingolfsson et al. [33], McLay [38], and Ansari et al. [39].

Finally, to overcome the limitation of assuming that the busy fractions are independent of the decisions made, many studies use an iterative procedure wherein the busy fractions are recalculated every time a new solution is obtained, after which the model is re-optimized using the revised busy fractions (e.g. Batta et al. [28], Ingolfsson et al. [33], Ansari et al. [39]).

Although many studies have focused on overcoming the MEXCLP’s simplifying assumptions, very few studies have considered applications such as CFR systems for which multiple of the MEXCLP’s assumptions are reasonable. Unlike a centrally-controlled fleet of ambulances, volunteers behave independently [10]. Moreover, their availability is externally determined and generally not impacted by decision-making in CFR systems because the probability that volunteers receive simultaneous alerts is generally very low [17]. This is in stark contrast to many EMS systems, where servers become unavailable due to being assigned calls. Hence, only the assumption of homogeneous busy fractions is unreasonable for CFR systems. Existing studies considering server-specific busy fractions typically also incorporate a hypercube model to account for server dependence. As such, these studies often resort to heuristic approaches due to the complexity of the corresponding problems. Only Ingolfsson et al. [33] optimally solve a MEXCLP formulation with server-specific busy fractions. However, only very small instances could be solved optimally with their optimization approach as shown by van den Berg et al. [40]. Therefore, we develop an optimization model that is mathematically equivalent to an expected coverage optimization problem with server-specific availability, as well as a corresponding solution approach that efficiently obtains optimal solutions for realistically-sized instances.

## Problem formulation

This section introduces the optimization problem of deciding how to train the volunteers of a CFR system under a limited budget to maximize the system’s effectiveness. We focus on CFR systems where volunteers are dispatched to multiple emergency types and classified based on their medical skills. Each class corresponds to a specific training level. A volunteer’s training level then fully determines the emergencies to which this volunteer can be dispatched. The higher the training level, the more medical skills the volunteer possesses and the more emergency types the volunteer can be dispatched to.

The optimization problem subsequently involves selecting which volunteers to train and to what level to maximize the effectiveness of the CFR system given a certain budget. We use the expected relief, defined as the probability that at least one volunteer arrives before the ambulance, to measure the effectiveness of CFR systems.

We have a set of volunteers *V* and a set of areas *A* where emergencies occur. Emergency types can have different severity levels, denoted by the set *S*, and different required training levels, denoted by the set *L*. The severity of an emergency defines how time-sensitive it is, while the required training level defines the medical skills a volunteer must have to be able to provide first aid. Emergency types with the same severity level can have different required training levels, and vice versa. An incident’s severity and required training level are not inherently correlated. For example, while OHCA is extremely time-sensitive, relatively few medical skills are required to provide CPR or apply an AED. We also incorporate a set of time intervals *I* to account for temporal patterns in volunteer availability and incident arrivals. We assume that incidents occur according to a Poisson process. Let $\lambda _{a,i,s,l}$ denote the arrival rate of incidents within area *a* during interval *i* of severity *s* with required training level *l*. Without loss of generality, we assume that the arrival rates sum up to 1.

Naturally, volunteers’ availability and whether they can reach incidents before the ambulance are both highly stochastic. Let $p_{v,a,s,i}$ be the probability that volunteer *v* is both available and able to reach an incident in area *a* of severity *s* before the ambulance during interval *i*. This probability can incorporate multiple sources of uncertainty, such as uncertainty in volunteer availability, volunteer location, and ambulance response times. This probability can be estimated in different ways depending on the available data. LIVES, for example, has data on when volunteers have been available in the past. Hence, this probability can be obtained by multiplying the probability that a volunteer is available by the probability that this volunteer would arrive before the ambulance for a specific incident when available. The latter can be estimated using both data on ambulance response times for historical incidents and information on volunteers’ locations, for example.

Let $z_{v,l}$ be a binary decision variable indicating if volunteer *v* has *at least* training level *l* after executing the training decisions. Then, for an incident in area *a* during interval *i* of severity *s* with required training level *l*, the expected relief can be calculated as

1 $$\begin{aligned} \mathbb {E}\left[ \text {Relief} \right]&= \mathbb {P}\text {(At least one volunteer arrives before the ambulance)} \nonumber \\&= 1 - \mathbb {P}\text {(No volunteer arrives before the ambulance)} \nonumber \\&= 1 - \prod _{v \in V: z_{v,l} = 1} (1-p_{v,a,s,i}) \nonumber \\&= 1 - \prod _{v \in V} (1-p_{v,a,s,i} \cdot z_{v,l}) \nonumber \\&= 1 - \prod _{v \in V} (1-p_{v,a,s,i})^{z_{v,l}}. \end{aligned}$$

Several assumptions are made within this expression. First, we assume that the probabilities with which volunteers can reach the incident before the ambulance are independent because volunteers generally behave autonomously [10]. To verify this assumption, we show that the availability of LIVES’ volunteers is generally independent in the Online supplement. The reliance on ambulance response times introduces some dependency in the probabilities with which volunteers arrive before the ambulance. However, the degree of dependency is likely to be limited as volunteers are only dispatched when close to an incident. Second, we assume that volunteers do not receive simultaneous alerts, since the probability of this is generally very low [17]. Third, it is implicitly assumed that multiple volunteers are dispatched to the same incident or that it is known which volunteers can arrive before the ambulance at the time of dispatching. This assumption is reasonable for CFR systems since almost always multiple volunteers are dispatched to a single incident and increasingly the real-time locations of volunteers are tracked, allowing for a reasonable estimate of which volunteers could arrive before the ambulance. Finally, we must have that $p_{v,a,s,i} < 1 \ \forall \ v \in V$ as otherwise Eq. 1 is undefined. However, this requirement is non-restrictive as $p_{v,a,s,i}$ equals 1 only if this volunteer is always available and always arrives before the ambulance, something which is highly unrealistic in practical applications and could otherwise easily be accounted for in the optimization model.

Equation 1 cannot be optimized directly as it is highly nonlinear with respect to the decision variables. Therefore, we rewrite Eq. 1 in the following way.

2 $$\begin{aligned} \mathbb {E}\left[ \text {Relief} \right]&= 1 - \prod _{v \in V} (1-p_{v,a,s,i})^{z_{v,l}} \nonumber \\&= 1 - \exp { \left( \ln { \left( \prod _{v \in V} (1-p_{v,a,s,i})^{z_{v,l}}\right) }\right) } \nonumber \\&= 1 - \exp { \left( \sum _{v \in V} \ln {\left( (1-p_{v,a,s,i})^{z_{v,l}}\right) }\right) } \nonumber \\&= 1 - \exp {\left( \sum _{v \in V} z_{v,l} \cdot \ln {(1-p_{v,a,s,i})}\right) }. \end{aligned}$$

It is required once more that $p_{v,a,s,i} <1 \ \forall \ v \in V$ as otherwise Eq. 2 is undefined.

Equation 2 specifies the expected relief for a specific area *a*, severity level *s*, required training level *l*, and interval *i*. The system-wide expected relief can be calculated as

3 $$\begin{aligned} f({\textbf {z}}) :&= \sum _{a \in A} \sum _{i \in I} \sum _{s \in S} \sum _{l \in L} \lambda _{a,i,s,l} \cdot \left( 1 - \prod _{v \in V} (1-p_{v,a,s,i})^{z_{v,l}} \right) \end{aligned}$$

4 $$\begin{aligned}&= \sum _{a \in A} \sum _{i \in I} \sum _{s \in S} \sum _{l \in L} \lambda _{a,i,s,l} \cdot \left( 1 - \exp { \left( \sum _{v \in V} z_{v,l} \cdot \ln {(1-p_{v,a,s,i})}\right) } \right) . \end{aligned}$$

It is easy to see that maximizing Eq. 4 is mathematically equivalent to minimizing

5 $$\begin{aligned} \sum _{a \in A} \sum _{i \in I} \sum _{s \in S} \sum _{l \in L} \lambda _{a,i,s,l} \cdot \exp { \left( \sum _{v \in V} z_{v,l} \cdot \ln {(1-p_{v,a,s,i})}\right) }. \end{aligned}$$

Maximizing the system-wide expected relief is thus mathematically equivalent to minimizing a weighted sum of exponential functions, which can be well approximated using piecewise linear approximation. Therefore, optimization solvers like Gurobi can directly optimize the system-wide expected relief by using Eq. 4.

To formulate the entire optimization problem, let $t_v$ be the starting training level of volunteer *v* and let $W_v:= \{l \in L: l \ge t_v\}$ be the levels volunteer *v* can be trained to, including the possibility to keep volunteer *v* at the current training level. Define $x_{v,l}$ for $v \in V, l \in W_v$ as the decision variable of whether volunteer *v* is trained to level *l*. Finally, let *B* denote the available training budget and let $c_{l_1,l_2}$ denote the cost of training a volunteer from level $l_1$ to level $l_2 \ge l_1$. The optimization problem can then be formulated as

**Maximize**

6 $$\begin{aligned} \sum _{a \in A} \sum _{i \in I} \sum _{s \in S} \sum _{l \in L} \lambda _{a,i,s,l} \cdot \left( 1 - \exp { \left( \sum _{v \in V} z_{v,l} \cdot \ln {(1-p_{v,a,s,i})}\right) } \right) \end{aligned}$$

**Subject to**

7 $$\begin{aligned} \sum _{l \in W_v} x_{v,l}&= 1 \quad & \forall v \in V \end{aligned}$$

8 $$\begin{aligned} \sum _{v \in V} \sum _{l \in W_v} x_{v,l} \cdot c_{t_v, l}&\le B \quad & \end{aligned}$$

9 $$\begin{aligned} z_{v, l}&= \sum _{l' \in W_v : l' \ge l} x_{v,l'} \quad & \forall v \in V, l \in L \end{aligned}$$

10 $$\begin{aligned} x_{v,l}&\in \{0,1\} \quad & \forall v \in V, l \in W_v \end{aligned}$$

11 $$\begin{aligned} z_{v,l}&\in \{0,1\} \quad & \forall v \in V, l \in L \end{aligned}$$

Constraint 7 ensures that exactly one training option is chosen for each volunteer. Constraint 8 enforces the budget constraint. Constraint 9 ensures the connection between the *x* and the *z* variables. Note that this constraint results from the hierarchical training structure. If desired, the model can be adapted to accommodate any arbitrary training structure without increasing problem complexity. We provide this modified optimization model in the Online supplement. Finally, Constraints 10 and 11 impose the integrality constraints of the decision variables. Note that a feasible solution to this problem always exists if maintaining a volunteer at the current training level does not incur any costs.

As mentioned, optimal solutions for realistically-sized instances can be obtained efficiently using piecewise linear approximation. Although using piecewise linear approximation might induce an approximation error, we provide a theoretical upper bound in the Online supplement showing that potential approximation errors are negligible.

One limitation of the optimization model is that it requires an optimization solver capable of solving nonlinear problems or large-scale linear problems approximating the nonlinear problem. Many CFR systems are operated by organizations with limited financial funds and are therefore unlikely to acquire such solvers. As an alternative, in the Online supplement, we present a different, more compact, linearized formulation that can be implemented with open-source solvers. Although this alternative formulation has a theoretically unbounded approximation error, we also provide a numerical analysis in the Online supplement showing that this alternative formulation provides near-optimal solutions very efficiently.

Finally, the introduced optimization model can also be used to optimize the expected coverage when defining $p_{v,a,s,i}$ to be the probability that volunteer *v* can cover an incident of severity *s* occurring in area *a* during interval *i*.

## Illustrative example

This section discusses an example to demonstrate the optimization approach and the difficulty of selecting which volunteers to train. Before discussing the example, however, it is important to highlight that three characteristics of volunteers generally determine the value of training them: their availability, the number of incidents occurring in their vicinity, and their ability to reach incidents that relatively few other volunteers can reach. This example illustrates that each characteristic can be most important for deciding which volunteers to train and that, for a specific instance, it is generally not clear which characteristic is most important.

We consider the choice of training one of three volunteers. These volunteers can only be dispatched to incidents when trained. Each volunteer is located in a distinct area and can arrive before the ambulance only for incidents in this area. We assume that volunteers always arrive before the ambulance in their respective areas when dispatched; however, as long as all three volunteers have the same probability of arriving before the ambulance, this probability does not affect the outcomes of the example. For simplicity, time intervals and severity levels are not considered.

The input parameters are provided in Table 1. The areas already have a non-zero expected relief due to other trained volunteers. As can be seen, each volunteer stands out with respect to one of the three abovementioned characteristics.

**Table 1:** Input parameters of the illustrative example. The arrival rate and current expected relief hold for the area in which the corresponding volunteer is located

	Availability	Arrival rate	Current expected relief
Volunteer 1	10%	100	0.5
Volunteer 2	20%	45	0.5
Volunteer 3	10%	45	0.1

The system-wide expected relief before training one of the three volunteers, which is the weighted average of the expected relief across all areas, equals $\frac{100\cdot 0.5+45\cdot 0.5+45\cdot 0.1}{100+45+45} \approx 0.405$, meaning that at least one volunteer will arrive before the ambulance in roughly 40% of all incidents. Training Volunteer 1 increases the expected relief of the corresponding area to $1-(1-0.5)\cdot (1-0.1) = 0.55$, which in turn increases the system-wide expected relief to $\frac{100\cdot 0.55+45\cdot 0.5+45\cdot 0.1}{100+45+45} \approx 0.432$. Similar calculations yield that the system-wide expected relief increases to 0.429 or 0.427 when training Volunteer 2 or 3, respectively. Hence, it is optimal to train Volunteer 1. The high arrival rate of the area in which Volunteer 1 is located is thus the most important determinant of the training decision.

However, a different conclusion arises when the input of the instance changes slightly. Consider the scenario in which the arrival rates of all three areas increase by 15 (i.e., the arrival rates become 115, 60, and 60). It would no longer be optimal to train Volunteer 1. Instead, performing similar calculations as above yields that it is optimal to train Volunteer 2, indicating that the higher availability of Volunteer 2 is the most important determinant of the training decision in this scenario.

Now consider the scenario in which the expected relief provided by existing volunteers is 0.2 higher for all three areas compared to the baseline (i.e., the current expected reliefs are 0.7, 0.7, and 0.3). It is optimal to train Volunteer 3 in this scenario. Therefore, the low expected relief of the area in which Volunteer 3 is located is the most important determinant of the training decision in this case.

Even for a highly simplified case, it is thus not immediately clear which volunteer should be trained to maximize the CFR system’s effectiveness, highlighting the need for an optimization approach to make informed training decisions.

## Case description

This section provides the details of the case study performed in collaboration with LIVES. LIVES is a charity providing first aid for medical emergencies within Lincolnshire, United Kingdom [15]. Lincolnshire is a primarily rural area of approximately 7,000 km$^2$ with a population of 1.1 million, yielding an average density of roughly 150 people per km$^2$. One of the main ways LIVES provides first aid is through its CFR system. LIVES’ CFRs are dispatched to various emergency types, such as strokes, hemorrhages, traffic accidents, and traumatic emergencies. The volunteers collectively provide medical assistance for 15 to 20 incidents per day on average.

LIVES’s volunteers have various medical backgrounds, ranging from individuals with a medical profession to individuals without any medical skills prior to becoming a CFR. The volunteers are classified into 8 classes corresponding to 8 different training levels. The higher the level of a class, the more medical skills volunteers within this class have, and the more emergency types they can be dispatched to. Due to having relatively few medical skills, volunteers with training level 1 can only be dispatched when at least one volunteer of level 2 or higher is also dispatched. Hence, in this case study, volunteers of level 1 cannot provide relief to any incident unless they are trained to a higher level.

LIVES trains its volunteers up to level 4 internally [41]. Although volunteers can be trained to level 5 or higher, this requires substantial external training and is very rare. Volunteers of level 5 or higher are almost exclusively off-duty medical professionals who already had extensive medical skills when becoming a CFR. Therefore, we only consider training volunteers up to level 4.

LIVES’ volunteers are committed to being available for at least 16 hours per month [42]. This is a minimum requirement, with many volunteers being available much more. When available, a volunteer is expected to go to an incident when alerted. Volunteers communicate their availability directly to the dispatch center, which is under the control of the ambulance control center. Note that this causes only the real-time availability to be deterministic; the long-term availability of volunteers remains highly uncertain and requires the probabilistic modeling approach introduced previously. Volunteers determine themselves which incidents in terms of severity they are available for. Emergency types are classified into three different severity levels (i.e., $S:= \{1,2,3\}$). Severity levels 1, 2, and 3 represent high, medium, and low time-sensitive incidents, respectively. Volunteers can indicate that they are available only for incidents of severity 1, only for incidents of severity 1 or 2, or for all incidents. This is because volunteers may not want to be dispatched to less severe incidents to keep the impact on their personal lives acceptable.

The volunteers also communicate their real-time location to the dispatcher. Volunteers have a fixed base location from which they respond most often, but they inform the dispatcher when they respond from a different location. The dispatcher thus knows which volunteers are available and their locations at any point in time. When an incident occurs near an available volunteer, the dispatcher can alert this volunteer if the volunteer has at least the required training level, the incident is declared safe, and the volunteer is within 10 kilometers of the incident. Although volunteers have the right to decline an alert, this is extremely rare. Hence, we assume that volunteers always go to the incident when dispatched. Although this is a strong assumption for CFR systems in general, it is reasonable for LIVES since dispatchers only reach out to available volunteers and are very rarely declined.

### Data description and parameter estimation

LIVES has provided historical data on incidents to which at least one volunteer has been dispatched, as well as volunteer-specific data such as their availability over time, training levels, and base locations. LIVES also provided the costs of training volunteers from level $l_1$ to level $l_2$ as well as the yearly training budget. Keeping volunteers at their current training levels does not incur any costs.

The incident data spans from April 2020 until November 2023 and includes 21,755 incidents. The incident data contains information on the emergency type, location, and timestamps on the emergency call time, CFR dispatch time, and ambulance arrival time. We exclude 1,396 incidents due to either missing information or occurring outside Lincolnshire. The incident data has been anonymized by aggregating incident locations per postal code.

The volunteer availability data spans from January 2022 until November 2023 and includes 238 volunteers having 13,205 sessions collectively, where each session represents one uninterrupted period during which a specific volunteer was available. For each session, the data specifies the start and end times of this period and for which severity levels the volunteer was available. We exclude 46 volunteers who have fewer than five sessions or for whom the current training level or base location is unknown, as the input parameters cannot be estimated accurately for these volunteers. The remaining 192 volunteers collectively have 13,006 sessions in the availability data. The average duration of a session is approximately 9.5 hours. The availability of volunteers can differ substantially: whereas the average probability of being available is 0.052 across all volunteers, the volunteer with the highest availability is available more than half of the time.

Both incident arrivals and volunteer availability display strong daily patterns. They are lowest during nighttime, highest in the evening, and at intermediate levels during daytime. We use three different intervals to capture these patterns, which span from 00:00 until 09:00, from 09:00 until 17:00, and from 17:00 until 00:00. No distinction for the day of the week is made since neither incident arrivals nor volunteer availability displayed substantial differences. Using three intervals reduced the variability within each interval in terms of arrival rates and volunteer availability levels considerably compared to using a single interval. Additionally, the intervals do not contain any systematic patterns, such as some volunteers always being available at the beginning of an interval and some volunteers always being available at the end of an interval. Although using more than three intervals marginally further decreased the variance within intervals, we decided not to use more than three intervals to maintain sufficient data per parameter and limit the risk of overfitting.

We use the 2021 Middle Layer Super Output Areas (MSOAs) within Lincolnshire as the set of areas [43]. MSOAs are areas used for reporting census statistics and are constructed to generally have a population between 5,000 and 15,000 [44]. There are 133 MSOAs within Lincolnshire.

We combine the incident data with population statistics to estimate the arrival rates. This is because the incident data contains a selection bias as it does not include incidents for which no volunteer was dispatched. Hence, we use population statistics to calculate the total arrival rate per area and use the incident data to determine the distributions with respect to the emergency types and time intervals. A detailed explanation of the estimation process is provided in the Online supplement.

To estimate the relief probabilities (i.e., the probabilities with which volunteers arrive before the ambulance), we distinguish between uncertainty in volunteer availability and uncertainty in whether volunteers can arrive before the ambulance when available. Let $p_{v,i,s}^1$ be the probability that volunteer *v* is available to be dispatched to incidents of severity *s* during interval *i* and let $p_{v,a,s}^2$ be the probability with which volunteer *v* arrives before the ambulance for an incident of severity *s* within area *a* when available. Then, assuming that both sources of uncertainty are independent, the relief probabilities can be calculated as

$$\begin{aligned} p_{v,a,s,i} = p_{v,i,s}^1 \cdot p_{v,a,s}^2. \end{aligned}$$

The parameters $p_{v,i,s}^1$ are estimated as the historical probability that volunteer *v* was available to respond to an incident of severity *s* during interval *i*. We do not consider the possibility of volunteers receiving simultaneous alerts as the probability of this happening is generally very low [17]. By letting the availability probabilities depend on the severity levels, we directly account for the possibility that volunteers are available only for highly urgent incidents. Note that these availability probabilities are independent of the training decisions because there is no inherent correlation between the severity level and the required training level of an incident. The parameters $p_{v,a,s}^2$ are estimated as the fraction of historical incidents in area *a* of severity *s* for which volunteer *v* could have arrived before the ambulance. These parameters account for the fact that volunteers could only have arrived before the ambulance when they were within 10 kilometers of the incident, as otherwise they would not have been dispatched. Hence, a single volunteer *v* will have relatively few non-zero $p_{v,a,s}^2$ parameters in practice. The Online supplement provides a more detailed explanation of how the parameters are estimated. Finally, for each volunteer *v* the set $W_v$ can be determined directly based on the current training levels of the volunteer.

**Fig. 1:**
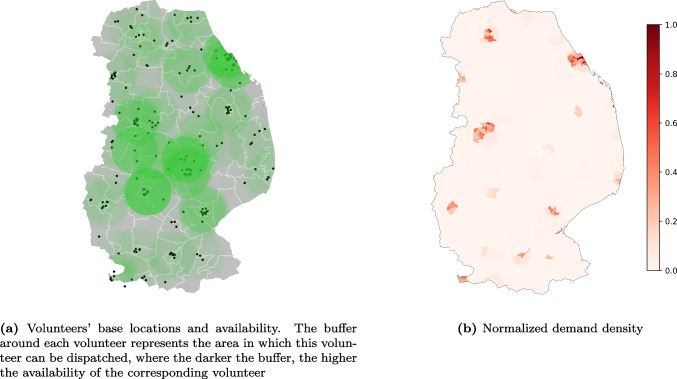
Volunteer availability and normalized demand density

The volunteers’ availability and normalized demand densities are shown in Fig. 1a and b. These figures highlight the large geographical differences in volunteer availability and demand density, underlining the importance of accounting for these geographical differences.

### Alternative training strategies

We also analyze three intuitive training strategies that likely resemble approaches adopted in practice more closely. Each strategy focuses on one specific important characteristic of volunteers as discussed previously: their availability, the number of incidents occurring in their vicinity, or their ability to reach incidents that relatively few other volunteers can reach. These three training strategies greedily select volunteers until there is no budget left. Specifically, the three strategies work as follows.

- The *availability heuristic* greedily selects the volunteer with the highest average probability of being available.
- The *demand heuristic* greedily selects the volunteer with the most past incidents that have occurred within 10 km, where ties are broken based on volunteer availability.
- The *remoteness heuristic* greedily selects the volunteer for which the incidents that have occurred within 10 km have the smallest average number of other volunteers located within 10 km. Again, ties are broken based on volunteer availability.

## Numerical results

This section presents the numerical results of the case study. We used the optimization approach based on piecewise linear approximation to obtain the optimal solutions. All computations were performed on an Intel(R) Core(TM) it-1265U laptop with 16GB of RAM using Gurobi 12.0.0 as the optimization solver.

### Optimizing a one-year budget

**Fig. 2:**
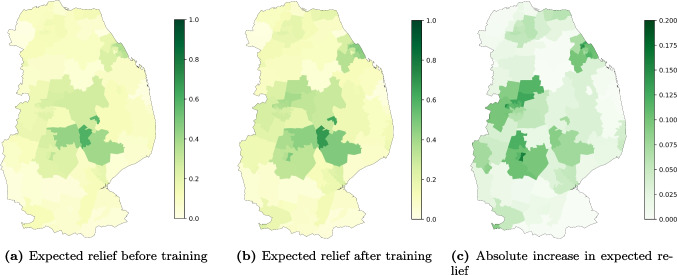
Results of the optimization model for a one-year budget

The system-wide expected relief is 0.169 before training any volunteers, meaning that at least one volunteer will arrive before the ambulance in approximately 17% of the incidents. The average initial expected relief (i.e., the average expected relief before training volunteers) varies considerably per area as shown in Fig. 2a. The median initial expected relief is 0.148, with values ranging from 0.008 to 0.594 (IQR = [0.088, 0.234]). The initial expected relief per area is positively associated with population density, having a correlation coefficient of 0.393.

Optimally spending a one-year budget increases the system-wide expected relief to 0.228, a 34.7% improvement. Training all volunteers to level 4, which would require 3.61 times the yearly budget, would increase the system-wide expected relief to 0.251. This implies that already 70% of the maximum expected relief increase can be obtained by spending a one-year budget optimally.

Figure 2c shows that the absolute increase in expected relief also varies considerably across areas. Whereas for some areas the average expected relief does not increase, for other areas the average expected relief increases by 0.159. The median increase is 0.054 (IQR = [0.020, 0.097]). Interestingly, the expected relief increase is also positively associated with population density, having a correlation coefficient of 0.489. This is especially surprising as volunteers in rural areas have a higher probability of arriving before the ambulance than volunteers in urban areas due to longer ambulance response times in rural areas. Three factors combined explain the positive correlation between the expected relief increase and population density.

First, ambulance response times in Lincolnshire are very high in general. For severity 2 incidents, which constitute the majority of all incidents, the average ambulance response time is almost one hour [45]. This results in all volunteers, even ones in urban areas, having a high chance of arriving before the ambulance because they are only dispatched to incidents in a 10-kilometer range. Even in the most densely populated areas, volunteers arrive before the ambulance in over 50% of all severity 1 incidents.

Second, volunteers in densely populated areas have far more incidents occurring within 10 kilometers than those in sparsely populated areas. Hence, the incentive to train volunteers in rural areas created by them having a higher probability of arriving before the ambulance is not large enough to compensate for the large differences in demand densities.

This is also evident from the fact that optimizing expected coverage using the same response-time targets as the National Health Service (NHS) yields the exact same optimal solution as optimizing the expected relief (the full results for coverage optimization are provided in the Online supplement). Hence, although optimizing expected relief explicitly accounts for ambulance response times - unlike expected coverage, which considers the CFR system in isolation - the impact of the ambulance response times is not large enough to lead to different optimal training decisions. However, note that this is not necessarily undesirable as it implies that there is no trade-off between improving coverage or relief because both can be improved simultaneously.

Third, although the initial expected relief is higher in densely populated areas than in sparsely populated areas, the average initial expected relief is relatively low in all areas for incidents that require training level 4. As can be seen in Table 2, the average initial expected relief for incidents requiring level 4 is less than half of the initial expected relief for incidents requiring level 3. Hence, relatively large expected relief improvements could be obtained by training volunteers to level 4, even in densely populated areas. This argument is supported by the fact that all volunteers selected to be trained in the optimal solution are trained to level 4 as shown in Table 3.

**Table 2:** Average expected relief per training level

	Level 2	Level 3	Level 4
Expected relief before training	0.249	0.193	0.091
Expected relief after training	0.251	0.231	0.208

These three factors combined make it optimal to train relatively many volunteers in densely populated areas. Therefore, the expected relief increases more in densely populated areas than in sparsely populated areas, despite their higher initial expected relief and shorter ambulance response times.

**Table 3:** Contingency table of old training levels (rows) and new training levels (columns)

	Level 1	Level 2	Level 3	Level 4	Level 5 or higher
Level 1	0	0	0	1	0
Level 2	0	48	0	15	0
Level 3	0	0	48	22	0
Level 4	0	0	0	31	0
Level 5 or higher	0	0	0	0	27

Recall that the value of training volunteers mainly depends on the number of incidents they can reach before the ambulance, their ability to reach incidents that relatively few other volunteers can reach, and their availability. The fact that trained volunteers are mainly located in densely populated areas suggests that trained volunteers can reach relatively many incidents before the ambulance, but not necessarily incidents that relatively few other volunteers can reach before the ambulance. Indeed, when available, trained volunteers could reach 6.9% of all incidents before the ambulance on average, whereas this is only 3.7% for untrained volunteers (i.e., volunteers of level 3 or lower who are not selected to be trained in the optimal solution). Moreover, incidents that trained volunteers can reach before the ambulance have an average initial expected relief of 0.204, whereas this is 0.169 on average for incidents that untrained volunteers can reach before the ambulance. The largest difference between trained and untrained volunteers, however, regards their availability. Trained volunteers are available 7.7% of the time on average, whereas this is only 2.3% for untrained volunteers. Therefore, the availability of volunteers is the most important determinant of the training decisions in this case study, followed by the number of incidents occurring near a volunteer.

### Performance of the alternative training strategies

**Fig. 3:**
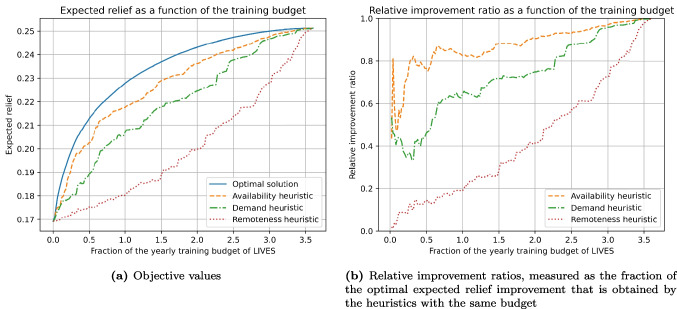
Performance of the alternative training strategies

Figure 3a displays the expected relief of the solutions obtained by the alternative training strategies as a function of the training budget. Before analyzing the alternative training strategies’ performance, however, note that the expected relief is not a non-decreasing function of the training budget for these strategies. This results from a misalignment between the selection and evaluation metrics. The alternative training strategies represent intuitive approaches that select volunteers based on one of their three desirable characteristics and not on the expected relief increase. Therefore, when different trainings have different costs, as is the case in this case study, increasing the training budget can lead to another volunteer being trained, who yielded a lower expected relief increase despite having a better value on the selection metric. Moreover, note that the marginal expected relief increase as a result of more budget being available generally decreases for the optimal solution. This is because the optimization model selects the volunteers enabling the greatest increase in the expected relief for a given budget. When training a specific set of volunteers, the potential increase in the expected relief resulting from training a different volunteer cannot increase, causing the marginal expected relief increase resulting from more budget being available to be broadly decreasing.

When analyzing the performance of the alternative training strategies, there is a clear order in performance. The *availability heuristic* performs best, the *demand heuristic* performs second best, and the *remoteness heuristic* performs worst. The distance to the optimal objective value is typically twice as large for the demand heuristic as for the availability heuristic, whereas this is even more than four times as large for the remoteness heuristic. Although the performance of the alternative training strategies is roughly similar when 3.5 times the yearly budget is used, this is because nearly all volunteers can be fully trained in this case, rendering the order in which volunteers are selected unimportant.

The Availability heuristic provides solutions for which the expected relief is typically within 0.01 of the optimal expected relief. Although this limited absolute difference might suggest near-optimal performance, analyzing how efficiently the different strategies spend the training budget shows that the performance difference is much larger. Figure 3b shows that the optimization model spends the training budget much more efficiently than the heuristics for a wide range of budgets. The increase in the expected relief is more than 15% lower for the Availability heuristic than for the optimization model when a one-year training budget is used, whereas this is 35% and 80% lower for the Demand heuristic and the Remoteness heuristic, respectively. This indicates that considering only a single characteristic of volunteers leads to an inefficient spending of the limited training budget, with the degree of inefficiency depending on the specific characteristic considered.

### Imposing additional dispatch restrictions

All volunteers selected to be trained are trained to level 4 in the optimal solution. Although the alert frequency of a single volunteer is typically low because volunteers are only alerted for incidents within 10 kilometers, this training strategy might increase the risk of overburdening volunteers. This is because the higher the training level of a volunteer, the more emergency types this volunteer could be dispatched to due to the hierarchical training structure. Therefore, higher-level volunteers will be dispatched more often than lower-level volunteers on average, all else being equal.

The dispatch center recognizes the risk of overburdening volunteers and already acts upon it by dispatching lower-level volunteers if multiple volunteers are available and sufficiently close. The optimization model allows for the analysis of the impact of similar dispatch restrictions. Specifically, by adjusting Constraint 9, it is possible to incorporate the restriction that volunteers can be dispatched only to incidents for which the required training level is at most *n* levels lower than their own. We have analyzed multiple scenarios with different values of *n* in the Online supplement. The results show that although the availability of volunteers remains the most important characteristic on which volunteers are selected in all scenarios, dispatch restrictions can have strong implications for how much, where, and to what level volunteers should be trained. It is thus important to take dispatch restrictions into account when training volunteers.

## Conclusion and discussion

CFR systems provide a relatively new but potentially very effective opportunity to reduce emergency response times, especially in rural areas where ambulances often take longer to arrive. We studied a new optimization problem relevant to CFR systems: the problem of selecting what training to provide to which volunteer to maximize a CFR system’s effectiveness given a certain budget. We measured the effectiveness of CFR systems by the expected relief: the probability that at least one volunteer arrives before the ambulance. We introduced a problem formulation that takes into account the unique and heterogeneous characteristics of volunteers, as well as important geographical differences. Additionally, we developed a solution approach that efficiently obtains optimal solutions for realistically-sized instances and that can also be used for expected coverage optimization problems with server-specific availability.

We subsequently used the optimization model to find the optimal training decisions for LIVES’ CFR system. The results show that training highly available volunteers in densely populated areas maximizes the effectiveness of LIVES’ CFR system. The availability of volunteers remains the most important characteristic upon which volunteers are selected even when dispatch restrictions are imposed; however, such dispatch restrictions have important implications for how much, where, and to what level volunteers should be trained. These results are specific to LIVES’ CFR system, which has some unique features, such as volunteers who only very rarely decline an alert when they have indicated that they are available. However, the optimization approach can be adapted rather easily to accommodate different CFR systems with different characteristics. For example, the relief probabilities are sufficiently generic to incorporate additional sources of uncertainty, such as uncertainty in whether volunteers accept alerts.

Besides these insights regarding the optimal training strategy, this work also resulted in several valuable practical insights. First and foremost, this work underscored the importance of high-quality data and the awareness of potential biases. Specifically, LIVES’ incident data did not contain any incidents for which no CFR was dispatched. This created a significant selection bias in the data, which needed to be accounted for when estimating the model parameters to provide an accurate representation of reality. With this insight, LIVES recently intensified its collaboration with the ambulance dispatch center to enhance data sharing, as well as its efforts to find CFR-specific patterns in data. Additionally, our search for suitable metrics to measure the effectiveness of a CFR system contributed to a renewed discussion within LIVES on the value of volunteers and the impact of their interventions.

There are several promising directions for future research. First, the main limitation of the optimization model is that it is designed to make one-shot decisions. In reality, training volunteers is a continuous process with regular training opportunities. Amongst others, this implies that several key inputs for the optimization model, such as the volunteer base, the availability of volunteers, and the incident arrivals, will likely change over time. Additionally, the long timescale complicates accurate parameter estimation and increases the risk of suboptimal out-of-sample performance. We sought to limit biases when estimating the arrival rates by combining the incident data with population statistics. However, the relatively short period covered by the availability data did not allow us to incorporate changing volunteer behavior, nor to analyze out-of-sample performance. As such, future research could extend the optimization approach to a multi-period setting and analyze out-of-sample performance. This could, for example, be done by incorporating the optimization approach into an iterative framework where the model is used every time new training decisions need to be made. This would allow for the opportunity to continuously act upon changes that occur gradually over time and to revise model parameters with the most recent data each time new training decisions are made, potentially decreasing model inaccuracies and the risk of poor out-of-sample performance caused by dynamics such as changing volunteer behavior. Alternatively, the optimization model could be extended to a multiple-period setting with period-specific budgets available for training volunteers. Such an approach could better coordinate volunteer training over multiple periods and allows for incorporating additional practical considerations, such as limiting the amount of training per volunteer per time period.

A second direction for future research would be to more explicitly balance the well-known efficiency-equity trade-off. Although optimizing the expected relief provides an explicit incentive to train volunteers in rural areas because these volunteers are more likely to arrive before the ambulance, this incentive was not large enough in the case study of LIVES to impact the training decisions; most trained volunteers were located in urban areas, thereby increasing rather than decreasing inequalities between urban and rural areas. If one were to design a solution more favorable to rural regions, this would result in reduced effectiveness - a phenomenon known as the price of fairness [46]. The magnitude of this price can roughly be estimated from the gap between the optimal solution and the remoteness heuristic seen in Fig. 3a. Several approaches used for optimization problems in different EMS systems to better balance the efficiency-equity trade-off could also be extended to the optimization model presented in this study. Examples include using multi-objective optimization [11–13, 47, 48], modifying the objective to give more weight to worse-off areas [49], optimizing the objective for the worst-off area [50], optimizing some fairness metric such as the Gini coefficient [51] or the average Envy [52], or enforcing equity constraints [50].

Finally, future research could focus on relaxing the assumption of independent servers. Although this assumption is more than reasonable for CFR systems, this is not the case for many other EMS systems. To the best of our knowledge, the optimization approach presented in this study is the first approach capable of solving realistically-sized instances for expected coverage optimization problems with server-specific busy fractions. An interesting extension of this research would be to extend the optimization approach to settings with server dependency. Doing so would not only make the optimization approach much more applicable for other EMS dsystems, but would also make it possible to study how coordinating volunteer availability impacts a CFR system’s effectiveness, for example.

## Supplementary Information

Below is the link to the electronic supplementary material.

Supplementary file 1 (pdf 3595 KB)

## Data Availability

The incident and volunteer data used in the case study is owned by LIVES and therefore cannot be shared publicly. The shapefiles defining the areas used and the corresponding population statistics are publicly accessible and are provided by the Office for National Statistics under the Open Government License.

## References

[1] Caputo ML, Muschietti S, Burkart R, Benvenuti C, Conte G, Regoli F, Mauri R, Klersy C, Moccetti T, Auricchio A (2017) Lay persons alerted by mobile application system initiate earlier cardio-pulmonary resuscitation: a comparison with sms-based system notification. Resuscitation 114:73–78. 10.1016/j.resuscitation.2017.03.003 28268186 10.1016/j.resuscitation.2017.03.003

[2] Berglund E, Claesson A, Nordberg P, Djärv T, Lundgren P, Folke F, Forsberg S, Riva G, Ringh M (2018) A smartphone application for dispatch of lay responders to out-of-hospital cardiac arrests. Resuscitation 126:160–165. 10.1016/j.resuscitation.2018.01.039 29408717 10.1016/j.resuscitation.2018.01.039

[3] Andelius L, Malta Hansen C, Lippert FK, Karlsson L, Torp-Pedersen C, Kjær Ersbøll A, Køber L, Collatz Christensen H, Blomberg SN, Gislason GH, Folke F (2020) Smartphone activation of citizen responders to facilitate defibrillation in out-of-hospital cardiac arrest. J Am Coll Cardiol 76(1):43–53. 10.1016/j.jacc.2020.04.073 32616162 10.1016/j.jacc.2020.04.073

[4] Valeriano A, Van Heer S, de Champlain F, Brooks SC (2021) Crowdsourcing to save lives: a scoping review of bystander alert technologies for out-of-hospital cardiac arrest. Resuscitation 158:94–121. 10.1016/j.resuscitation.2020.10.035 33188832 10.1016/j.resuscitation.2020.10.035

[5] Folke F, Andelius L, Gregers MT, Malta Hansen C (2021) Activation of citizen responders to out-of-hospital cardiac arrest. Curr Opin Crit Care 27(3):209–215. 10.1097/MCC.0000000000000818 33769420 10.1097/MCC.0000000000000818

[6] Botan V, Asghar Z, Rowan E, Smith MD, Patel G, Phung VH, Trueman I, Spaight R, Brewster A, Mountain P, Orner R, Siriwardena AN (2023) Community first responders’ contribution to emergency medical service provision in the united kingdom. Ann Emerg Med 81(2):176–183. 10.1016/j.annemergmed.2022.05.025 35940990 10.1016/j.annemergmed.2022.05.025

[7] McLay LA, Mayorga ME (2010) Evaluating emergency medical service performance measures. Health Care Manag Sci 13:124–136. 10.1007/s10729-009-9115-x 20629415 10.1007/s10729-009-9115-x

[8] Lapidus O, Jonsson M, Svensson L, Hollenberg J, Berglund E, Riva G, Claesson A, Nordberg P, Rosenqvist M, Forsberg S, Nord A, Ringh M (2023) Effects of a volunteer responder system for out-of-hospital cardiac arrest in areas of different population density-a retrospective cohort study. Resuscitation 191:109921. 10.1016/j.resuscitation.2023.109921 37543160 10.1016/j.resuscitation.2023.109921

[9] Kragh AR, Gregers MT, Andelius L, Grabmayr AJ, Kollander L, Kjærulf VE, Kjølbye JS, Sheikh AP, Ersbøll AK, Folke F, Malta Hansen C (2024) Volunteer responder interventions in out-of-hospital cardiac arrest in urban, suburban, and rural areas. J Am Heart Assoc 13(4):e032629. 10.1161/JAHA.123.032629 38348801 10.1161/JAHA.123.032629 PMC11010116

[10] Paz JC, Kong N, Lee S (2022) Incorporating real-time citizen responder information to augment ems logistics operations: a simulation study. Comput Ind Eng 171:108399. 10.1016/j.cie.2022.108399

[11] Enayati S, Mayorga ME, Toro-Díaz H, Albert LA (2019) Identifying trade-offs in equity and efficiency for simultaneously optimizing location and multipriority dispatch of ambulances. Int Trans Oper Res 26(2):415–438. 10.1111/itor.12590

[12] Grot M, Nagel L, Becker T, Fiebrandt PM, Werners B (2022) Fairness or efficiency-managing this conflict in emergency medical services location planning. Comput Ind Eng 173:108664. 10.1016/j.cie.2022.108664

[13] Chanta S, Mayorga ME, McLay LA (2014) Improving emergency service in rural areas: a bi-objective covering location model for ems systems. Ann Oper Res 221:133–159. 10.1007/s10479-011-0972-6

[14] Gurobi (2025) Gurobi. https://www.gurobi.com/. Accessed 10 Apr 2025

[15] LIVES (2024) LIVES. https://www.lives.org.uk/. Accessed 31 Jul 2024

[16] Scquizzato T, Belloni O, Semeraro F, Greif R, Metelmann C, Landoni G, Zangrillo A (2022) Dispatching citizens as first responders to out-of-hospital cardiac arrests: a systematic review and meta-analysis. Eur J Emerg Med 29(3):163–172. 10.1097/MEJ.0000000000000915 35283448 10.1097/MEJ.0000000000000915

[17] van den Berg PL, Henderson SG, Jagtenberg CJ, Li H (2024) Modeling the impact of community first responders. Manage Sci 71(2):992–1008. 10.1287/mnsc.2022.04024

[18] Henderson SG, van den Berg PL, Jagtenberg CJ, Li H (2022) How should volunteers be dispatched to out-of-hospital cardiac arrest cases? Queueing Syst 100(3):437–439. 10.1007/s11134-022-09752-z

[19] Matinrad N, Andersson Granberg T, Angelakis V (2021) Modeling uncertain task compliance in dispatch of volunteers to out-of-hospital cardiac arrest patients. Comput Ind Eng 159:107515. 10.1016/j.cie.2021.107515

[20] Matinrad N, Andersson Granberg T (2023) Optimal pre-dispatch task assignment of volunteers in daily emergency response. Socioecon Plann Sci 87:101589. 10.1016/j.seps.2023.101589

[21] Slaa G (2020) Increasing cardiac arrest survival by improving the volunteer alerting algorithm. Master’s thesis, University of Twente

[22] Brotcorne L, Laporte G, Semet F (2003) Ambulance location and relocation models. Eur J Oper Res 147(3):451–463. 10.1016/S0377-2217(02)00364-8

[23] Li X, Zhao Z, Zhu X, Wyatt T (2011) Covering models and optimization techniques for emergency response facility location and planning: a review. Math Methods Oper Res 74:281–310. 10.1007/s00186-011-0363-4

[24] Farahani RZ, Asgari N, Heidari N, Hosseininia M, Goh M (2012) Covering problems in facility location: a review. Comput Ind Eng 62(1):368–407. 10.1016/j.cie.2011.08.020

[25] Bélanger V, Ruiz A, Soriano P (2019) Recent optimization models and trends in location, relocation, and dispatching of emergency medical vehicles. Eur J Oper Res 272(1):1–23. 10.1016/j.ejor.2018.02.055

[26] Church R, ReVelle C (1974) The maximal covering location problem. Pap Reg Sci 32(1):101–118. 10.1111/j.1435-5597.1974.tb00902.x

[27] Daskin MS (1983) A maximum expected covering location model: formulation, properties and heuristic solution. Transp Sci 17(1):48–70. 10.1287/trsc.17.1.48

[28] Batta R, Dolan JM, Krishnamurthy NN (1989) The maximal expected covering location problem: Revisited. Transp Sci 23(4):277–287. 10.1287/trsc.23.4.277

[29] Grot M, Becker T, Steenweg PM, Werners B (2022) Enhanced coverage by integrating site interdependencies in capacitated ems location models. Health Care Manag Sci 25(1):42–62. 10.1007/s10729-021-09562-4 34255237 10.1007/s10729-021-09562-4 PMC8983527

[30] Goldberg J, Dietrich R, Chen JM, Mitwasi MG, Valenzuela T, Criss E (1990) Validating and applying a model for locating emergency medical vehicles in tuczon, az. Eur J Oper Res 49(3):308–324. 10.1016/0377-2217(90)90402-W

[31] Goldberg J, Paz L (1991) Locating emergency vehicle bases when service time depends on call location. Transp Sci 25(4):264–280. 10.1287/trsc.25.4.264

[32] Saydam C, Aytuğ H (2003) Accurate estimation of expected coverage: revisited. Socioecon Plann Sci 37(1):69–80. 10.1016/S0038-0121(02)00004-6

[33] Ingolfsson A, Budge S, Erkut E (2008) Optimal ambulance location with random delays and travel times. Health Care Manag Sci 11:262–274. 10.1007/s10729-007-9048-1 18826004 10.1007/s10729-007-9048-1

[34] Larson RC (1974) A hypercube queuing model for facility location and redistricting in urban emergency services. Comput Oper Res 1(1):67–95. 10.1016/0305-0548(74)90076-8

[35] Larson RC (1975) Approximating the performance of urban emergency service systems. Oper Res 23(5):845–868. 10.1287/opre.23.5.845

[36] Jarvis JP (1985) Approximating the equilibrium behavior of multi-server loss systems. Manage Sci 31(2):235–239. 10.1287/mnsc.31.2.235

[37] Budge S, Ingolfsson A, Erkut E (2009) Approximating vehicle dispatch probabilities for emergency service systems with location-specific service times and multiple units per location. Oper Res 57(1):251–255. 10.1287/opre.1080.0591

[38] McLay LA (2009) A maximum expected covering location model with two types of servers. IIE Trans 41(8):730–741. 10.1080/07408170802702138

[39] Ansari S, McLay LA, Mayorga ME (2017) A maximum expected covering problem for district design. Transp Sci 51(1):376–390. 10.1287/trsc.2015.0610

[40] van den Berg PL, Kommer GJ, Zuzáková B (2016) Linear formulation for the maximum expected coverage location model with fractional coverage. Oper Res Health Care 8:33–41. 10.1016/j.orhc.2015.08.001

[41] LIVES (2024) LIVES, expert training. https://training.lives.org.uk/. Accessed 31 Jul 2024

[42] LIVES (2024) Emergency first responders. URL https://www.lives.org.uk/what-we-do/emergency-responders/emergency-first-responders/. Accessed 31 Jul 2024

[43] Office for National Statistics (2021) Middle layer Super Output Areas (December 2021) Boundaries EW BFC (V7). https://geoportal.statistics.gov.uk/datasets/12baf1e6a44441208ffe5ba5ed063a68_0/explore?location=52.715964%2C-2.489483%2C6.92. Accessed 6 Sept 2024

[44] Office for National Statistics (2021) Census 2021 geographies. https://www.ons.gov.uk/methodology/geography/ukgeographies/censusgeographies/census2021geographies. Accessed 6 Sept 2024

[45] Lincolnshire Live (2024) Lincolnshire ambulance service improves waiting times though concerns remain. https://www.lincolnshirelive.co.uk/news/local-news/lincolnshire-ambulance-service-improves-waiting-9057608. Accessed 27 Mar 2025

[46] Bertsimas D, Farias VF, Trichakis N (2011) The price of fairness. Oper Res 59(1):17–31. 10.1287/opre.1100.0865

[47] Gunnarsson B, Björnsdóttir KM, Dúason S, Ingólfsson A (2023) Locating helicopter ambulance bases in Iceland: efficient and fair solutions. Scand J Trauma Resuscit Emerg Med 31:70. 10.1186/s13049-023-01114-9 10.1186/s13049-023-01114-9 PMC10621180 37915061

[48] Luo W, Yao J, Mitchell R, Zhang X, Li W (2022) Locating emergency medical services to reduce urban-rural inequalities. Socioecon Plann Sci 84:101416. 10.1016/j.seps.2022.101416

[49] Jagtenberg CJ, Vollebergh MAJ, Uleberg O, Røislien J (2021) Introducing fairness in Norwegian air ambulance base location planning. Scand J Trauma Resuscit Emergency Med 29:50. 10.1186/s13049-021-00842-0 10.1186/s13049-021-00842-0 PMC7980553 33743747

[50] Akdogan MA, Bayındır ZP, Iyigun C (2023) An analysis of ambulance location problem from an equity perspective. Socioecon Plann Sci 90:101737. 10.1016/j.seps.2023.101737

[51] Toro-Díaz H, Mayorga ME, McLay LA, Rajagopalan HK, Saydam C (2015) Reducing disparities in large-scale emergency medical service systems. J Oper Res Soc 66(7):1169–1181. 10.1057/jors.2014.83

[52] Chanta S, Mayorga ME, Kurz ME, McLay LA (2011) The minimum p-envy location problem: a new model for equitable distribution of emergency resources. IIE Trans Healthcare Syst Eng 1(2):101–115. 10.1080/19488300.2011.609522

