# Supplementary material for: Ahead of the ambulance: Optimizing volunteer training
Source: Health Care Manag Sci. 2026 Jun 11;29(2):25. doi: 10.1007/s10729-026-09771-9 (PMC13253763; doi:10.1007/s10729-026-09771-9)
Supplement: Supplementary file 1 — (pdf 3595 KB) [file 10729_2026_9771_MOESM1_ESM.pdf]

Online supplement to "Ahead of the ambulance: Optimizing volunteer training"

A Independence of volunteer availability

In this section, we analyze any potential correlation in the availability of LIVES' volunteers to verify the assumption of volunteer availability being independent. To do so, we first calculate for all pairs of volunteers whether there is any overlap in the periods during which these volunteers were active (i.e. the period between the first and last moment a volunteer was available) using the availability data. Then, for every pair of volunteers for which the active periods overlapped at least one month, we calculate the correlation in their availability during this common active period per time interval. We use the same intervals as used in the case study (i.e. from 00:00 to 09:00, from 09:00 until 17:00, and from 17:00 until 00:00). For a specific pair of volunteers and a specific interval, we first create binary vectors for the availability of the two volunteers within this interval, where each entry represents one specific hour. These binary vectors are then used to calculate the Pearson correlation coefficient.

The distribution of all correlation coefficients is provided in Figure 1. The average correlation coefficient was found to be 0.005 (IQR = [-0.021, 0.0187]) with a standard deviation of 0.055. These results thus show that there is extremely little to no correlation in volunteer availability, supporting the assumption that volunteer availability is independent.

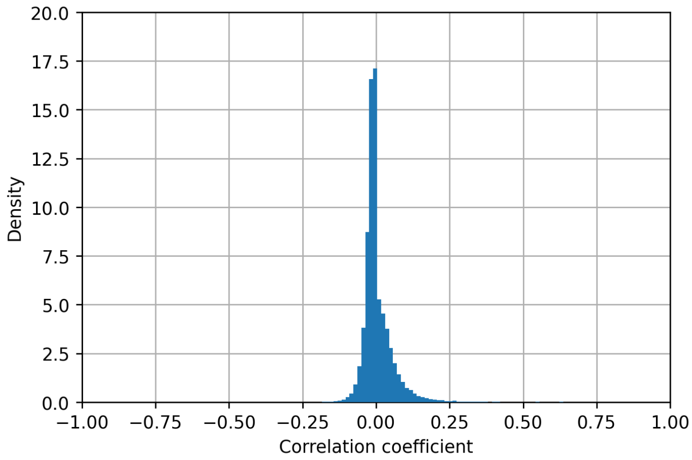

Figure 1: Correlation coefficients of the availability of volunteers

B Optimization model for arbitrary training structures

This section introduces the optimization model of maximizing a CFR system's effectiveness given a limited budget for systems with arbitrary training structures. The optimization model presented here relies on the same underlying assumptions as the model described in the main paper.

In line with the notation introduced in the main paper, let V be a set of volunteers, let A be a set of areas where incidents occur, let I be a set of time intervals, and let S be a set of severity levels. Additionally, let E denote a set of emergency types and let T be a set of trainings. We let a_{et} be a binary parameter indicating if training $t \in T$ covers emergency $e \in E$ (i.e., whether volunteers are allowed to be dispatched to incidents of this emergency type after having received this training). We let s_e denote the severity level of emergency type $e \in E$.

We define $s_{v,t}$ as a binary parameter indicating if volunteer $v \in V$ has training $t \in T$ from the beginning, $x_{v,t}$ as a binary decision variable if volunteer $v \in V$ receives training $t \in T$, and $z_{v,t}$ as a binary decision variable indicating if volunteer $v \in V$ has training $t \in T$ after executing the training decisions. Additionally, let $y_{v,e}$ be a binary decision variable indicating if volunteer $v \in V$ can respond to emergencies of type $e \in E$. Finally, let $p_{v,a,s,i}$ be the probability that volunteer v can reach an incident in area $a \in A$ of severity $s \in S$ before the ambulance during interval $i \in I$.

Then, the expected relief for an incident in area $a \in A$ during interval $i \in I$ of emergency type $e \in E$ can be derived similarly as done in the main paper. Specifically, the expected relief is given by

$$\begin{aligned}
\mathbb{E}\left[\text{Relief}\right] &= \mathbb{P}(\text{At least one volunteer arrives before the ambulance}) \\
&= 1 - \prod_{v \in V : y_{v,e} = 1} (1 - p_{v,a,s_e,i}) \\
&= 1 - \exp\left(\sum_{v \in V} y_{v,e} \cdot \ln\left(1 - p_{v,a,s_e,i}\right)\right).
\end{aligned}$$

To formulate the entire optimization model, let B denote the availabile training budget and let c_t denote the cost of providing training $t \in T$ to a single volunteer. Additionally, let $\lambda_{a,i,e}$ denote the arrival rate of incidents of emergency $e \in E$ within area $a \in A$ during interval $i \in I$. We assume without loss of generality that the arrival rates sum up to 1. The entire optimization model can then be stated as

Maximize

$$\sum_{a \in A} \sum_{i \in I} \sum_{e \in E} \lambda_{a,i,e} \cdot \left(1 - \exp\left(\sum_{v \in V} y_{v,e} \cdot \ln\left(1 - p_{v,a,s_e,i}\right)\right)\right) \tag{B.1}$$

Subject to

$$\sum_{v \in V} \sum_{t \in T} x_{v,t} \cdot c_t \leq B \tag{B.2}$$

$$z_{v,t} \leq s_{v,t} + x_{v,t} \qquad \forall v \in V, t \in T \tag{B.3}$$

$$y_{v,e} \leq \sum_{t \in T} a_{e,t} \cdot z_{v,t} \qquad \forall v \in V, e \in E \tag{B.4}$$

$$x_{v,t} \in \{0, 1\} \qquad \forall v \in V, t \in T \tag{B.5}$$

$$z_{v,t} \in \{0, 1\} \qquad \forall v \in V, t \in T \tag{B.6}$$

$$y_{v,e} \in \{0, 1\} \qquad \forall v \in V, e \in E \tag{B.7}$$

Objective B.1 maximizes the system-wide expected relief. Constraint B.2 enforces the budget constraint. Constraints B.3 and B.4 subsequently ensure the connection between the x, z, and y variables. Finally, Constraints B.5, B.6, and B.7 enforce the domain of the decision variables. Note that this optimization model can accommodate any arbitrary training structure as no assumptions were made regarding the a parameters.

C Upper bound on the approximation error

The main optimization approach introduced in this study relies on piecewise linear approximation to approximate the exact nonlinear objective of maximizing the expected relief. A small approximation error might occur due to this approach. Here, we provide a theoretical upper bound on this approximation error.

The functions approximated using piecewise linear approximation represent the probability that no single volunteer arrives before the ambulance (i.e. the complement of the expected relief). Gurobi allows controlling the maximum relative error of the piecewise linear approximation. Let ϵ denote the maximum relative error allowed. For a particular solution x, let $f(x)$ be the exact system-wide probability that no single volunteer arrives before the ambulance. The expected relief of this solution can thus be calculated as $1 - f(x)$. Similarly, let $f'(x)$ be the approximated system-wide probability that no single volunteer arrives before the ambulance. The approximated system-wide expected relief can then be calculated as $1 - f'(x)$. Moreover, let x^* denote the optimal solution according to the exact objective and let x' be the optimal solution according to the approximated objective. Due to the definition of ϵ, for any solution x we must have that

$$f'(x) \geq (1-\epsilon) \cdot f(x) \text{ or } f'(x) \cdot \frac{1}{1-\epsilon} \geq f(x), \tag{C.1}$$

and

$$f'(x) \leq (1+\epsilon) \cdot f(x) \text{ or } f'(x) \cdot \frac{1}{1+\epsilon} \leq f(x). \tag{C.2}$$

Consequently, we have that

$$\begin{aligned} f(x') &\leq \frac{1}{1-\epsilon} f'(x') && \text{(C.3)} \\ &\leq \frac{1}{1-\epsilon} f'(x^*) && \text{(C.4)} \\ &\leq \frac{1+\epsilon}{1-\epsilon} f(x^*). && \text{(C.5)} \end{aligned}$$

Here (C.3) follows from (C.1), (C.4) follows from the optimality of x', and (C.5) follows from (C.2). Note that (C.5) can also be written as $f(x^*) \geq \frac{1-\epsilon}{1+\epsilon} f(x')$. Using (C.5) it is possible to obtain an upper bound on the objective value of the optimal solution for the exact objective. This bound is given by

$$1 - f(x^*) \leq 1 - \frac{1-\epsilon}{1+\epsilon} f(x').$$

This bound can be used to obtain a lower bound on the quality of x', and an upper bound on the approximation error by extension. More specifically, the approximation error is bounded by

$$1 - \frac{1 - f(x')}{1 - f(x^*)} \leq 1 - \frac{1 - f(x')}{1 - \frac{1-\epsilon}{1+\epsilon} f(x')}.$$

Unfortunately, this bound is not constant in $f(x')$ (i.e. the exact system-wide probability that no volunteer arrives before the ambulance for the optimal solution of the approximated objective). However, this approximation error is decreasing in both $f(x')$ and ϵ as $\frac{1-\epsilon}{1+\epsilon} \leq 1$. The lowest possible maximum relative error supported by Gurobi is 10^{-6} (i.e. $\epsilon = 10^{-6}$). We use this lowest possible maximum relative error when solving the optimization model. The corresponding maximum approximation error is then extremely low, even for rather large values of $f(x')$. For example, the approximation error is upper bounded by 0.0002 if $f(x') = 0.99$ and upper bounded by 0.00002 if $f(x') = 0.9$.

D Alternative linear formulation

This section presents a linearized formulation of the optimization problem that can be used as an alternative to piecewise linear approximation. To obtain this linear formulation, first consider the following condition of whether the expected relief is at least some reliability level $\alpha \in (0, 1)$ for some tuple (a, i, s, l).

$$1 - \prod_{v \in V} (1 - p_{v,a,s,i})^{z_{v,l}} \geq \alpha.$$

This condition can be linearized in the following way.

$$\begin{aligned} 1 - \prod_{v \in V} (1 - p_{v,a,s,i})^{z_{v,l}} &\geq \alpha \\ 1 - \alpha &\geq \prod_{v \in V} (1 - p_{v,a,s,i})^{z_{v,l}} \\ \ln(1 - \alpha) &\geq \ln\left(\prod_{v \in V} (1 - p_{v,a,s,i})^{z_{v,l}}\right) \\ \ln(1 - \alpha) &\geq \sum_{v \in V} \ln\left((1 - p_{v,a,s,i})^{z_{v,l}}\right) \\ \ln(1 - \alpha) &\geq \sum_{v \in V} z_{v,l} \cdot \ln(1 - p_{v,a,s,i}). \end{aligned} \tag{D.1}$$

Condition (D.1) is linear in the decision variables as both α and $p_{v,a,s,i}$ are input parameters. Let $w_{a,i,s,l}$ be a binary variable indicating if the expected relief is at least $\alpha \in (0, 1)$ for some tuple (a, i, s, l). As both sides of Condition (D.1) are non-positive, Constraint (D.2) ensures that $w_{a,i,s,l}$ can only equal one if the expected relief is at least α.

$$\ln(1 - \alpha) \cdot w_{a,i,s,l} \geq \sum_{v \in V} z_{v,l} \cdot \ln(1 - p_{v,a,s,i}). \tag{D.2}$$

Constraint (D.2) is valid for only a single α. However, using only one reliability level is insufficient to calculate the expected relief. To obtain a linear expression of the expected relief, Constraint (D.2) has to be formulated for many reliability levels. Let $J := \{1, \ldots, n\}$ be the set of indices of n reliability levels and without loss of generality assume that $\alpha_j < \alpha_{j+1}$ for $j = 1, \ldots, n - 1$. Define $w_{a,i,s,l,j}$ as a binary variable indicating if the expected relief is at least α_j for some tuple (a, i, s, l). We define the objective coefficients in the following way to be able to obtain a linearized expression of the expected relief.

$$\omega_j = \begin{cases} \alpha_j & \text{if } j = 1, \\ \alpha_j - \alpha_{j-1} & \text{else.} \end{cases}$$

A linearized expression of the system-wide expected relief can then be formulated as

$$\sum_{a \in A} \sum_{i \in I} \sum_{s \in S} \sum_{l \in L} \lambda_{a,i,s,l} \cdot \left(\sum_{j \in J} \omega_j \cdot w_{a,i,s,l,j} \right). \tag{D.3}$$

The linearized expected relief given by (D.3) is always a lower bound of the exact expected relief due to the definition of ω_j. Enough reliability levels have to be used for the linearized expected relief given by (D.3) to be an accurate expression of the exact expected relief. The accuracy of the linearized expected relief as a function of the number of equidistant reliability levels is shown in Figure 2, where the optimal solution of the nonlinear integer program (NLIP) formulation for a one-year budget is used

to calculate the system-wide expected relief. As can be seen, although for a low number of equidistant reliability levels (e.g. $n \leq 10$) the accuracy is relatively low, it increases rapidly when n is increased.

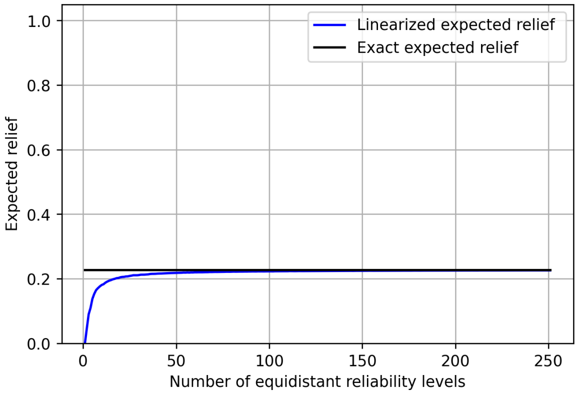

Figure 2: Accuracy of the linearized expected relief as a function of the number of equidistant reliability levels

The entire linearized formulation of the optimization problem can be specified as follows.

Maximize

$$\sum_{a\in A}\sum_{i\in I}\sum_{s\in S}\sum_{l\in L}\lambda_{a,i,s,l}\cdot\left(\sum_{j\in J}\omega_j\cdot w_{a,i,s,l,j}\right) \tag{D.4}$$

Subject to:

$$\ln(1-\alpha_j)\cdot w_{a,i,s,l,j} \geq \sum_{v\in V} z_{v,l}\cdot\ln(1-p_{v,a,s,i}) \quad \forall a\in A, s\in S, l\in L, i\in I, j\in J \tag{D.5}$$

$$\sum_{l\in W_v} x_{v,l} = 1 \quad \forall v\in V \tag{D.6}$$

$$\sum_{v\in V}\sum_{l\in W_v} x_{v,l}\cdot c_{t_v,l} \leq B \tag{D.7}$$

$$z_{v,l} = \sum_{l'\in W_v:l'\geq l} x_{v,l'} \quad \forall v\in V, l\in L \tag{D.8}$$

$$x_{v,l}\in\{0,1\} \quad \forall v\in V, l\in W_v \tag{D.9}$$

$$z_{v,l}\in\{0,1\} \quad \forall v\in V, l\in L \tag{D.10}$$

$$w_{a,i,s,l,j}\in\{0,1\} \quad \forall a\in A, s\in S, l\in L, i\in I, j\in J \tag{D.11}$$

Note that it would be feasible to have $w_{a,i,s,l,j}$ equal to 0 even if the expected relief is at least α_j for some tuple (a,i,s,l). However, this would not be optimal if $\omega_j > 0\ \forall\ j$, which holds if there are no duplicate reliability levels. If desired, however, it would be possible to force the w variables to 1 if the expected relief is at least α_j using big-M constraints.

Although for instances of moderate size it will generally take a long time for open-source solvers to obtain the optimal solution due to the number of constraints, near-optimal solutions can be obtained very efficiently. To demonstrate this, we have implemented this linearized formulation with HiGHS [2], an open-source solver that can be used to solve mixed integer linear programs. More specifically, we have solved the linear formulation presented above for $n = 1,\ldots,99$, where for each n the corresponding reliability levels are defined to be equidistant. We have imposed a time limit of 120 seconds when solving each model. The optimality gap of each solution with respect to the optimal solution of the NLIP formulation is shown in Figure 3. All computations were performed on an Intel(R) Core(TM) it-1265U laptop with 16GB of RAM.

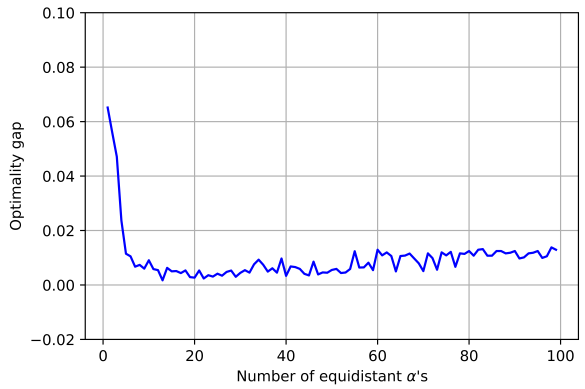

Figure 3: Optimality gaps of the solutions obtained by the linear formulation

As can be seen, the linearized formulation obtains near-optimal solutions very efficiently using an open-source solver, even for a low number of reliability levels. For example, when using only 20 reliability levels and imposing a time limit of 120 seconds, the linear model obtained a solution with an objective value only 0.27% lower than the objective value of the optimal solution of the NLIP formulation. Hence, despite the fact that when using a low number of reliability levels the linearized expected relief as defined by (D.4) is not yet an accurate expression of the exact (nonlinear) expected relief, the accuracy is sufficient to obtain near-optimal solutions very efficiently.

E Parameter estimation

This section provides a detailed explanation of how the arrival rates and relief probabilities are estimated in the case study performed in collaboration with LIVES.

We do not solely use the incident data provided by LIVES to estimate the arrival rates. This is because this data does not contain incidents to which no volunteer was dispatched, for example, due to no volunteer close enough being available at the time. Resultingly, there is a selection bias in the incident data. This selection bias mainly affects the total arrival rate per area and the distribution of incidents with respect to the required training level. To solve this, we use the number of inhabitants within an area as a proxy for the total number of incidents. The total arrival rate per area is therefore based on the fraction of inhabitants living within this area, where the 2021 MSOA population estimates as provided by Office for National Statistics [3] are used to determine the total inhabitants per area.

The distribution of incidents with respect to the required training level is based on which emergencies volunteers of a certain level can be dispatched to and the scope of practice provided by LIVES. Based on this information, we estimate that 30% of the incidents have level 2 as the required training level, 30% have level 3, and 40% have level 4. Note that no incident has level 1 as the required training level as volunteers of level 1 cannot be dispatched to incidents alone. Moreover, we disregard incidents requiring level 5 or higher since these incidents do not impact the optimal solutions. This is because we only consider training volunteers up to level 4 and assume that volunteers are not dispatched to simultaneous incidents.

For each area, the distribution of incidents with respect to the intervals and severity levels is based on past incidents in this area, as there does not necessarily exist a correlation between the severity and the required training level of an emergency. One difficulty is that not all areas contain incidents on which these distributions can be estimated. This is a result of incident locations being aggregated per postal code to ensure anonymity. Urban regions typically consist of more than one MSOA since the MSOAs are generated to have a population of roughly 5,000 to 15,000. However, there is generally only one incident location per urban region due to the aggregation of incident locations. This causes some areas not to contain any incidents, not because no incidents have occurred in this area, but because the incident locations have been aggregated. To resolve this, we utilize incident data from the nearest area containing recorded incidents to estimate the distribution of incidents in terms of severity levels and intervals for areas with no reported incidents. Here, the distance between areas is based on their respective centroids. This approach is used as areas without incidents are thus located in urban regions, and the closest area containing incidents is likely to be the most representative of this urban region.

In the case study, the relief probabilities are calculated as $p_{v,a,s,i} = p^1_{v,i,s} \cdot p^2_{v,a,s}$, where $p^1_{v,i,s}$ is the probability that volunteer v is available for incidents of severity s during interval i and $p^2_{v,a,s}$ is the probability with which volunteer v arrives before the ambulance for incidents of severity s within area a when available.

For every volunteer v, interval i, and severity level s, the probability $p^1_{v,i,s}$ can be calculated directly using the availability data.

For every volunteer v, area a, and severity level s, we calculate $p^2_{v,a,s}$ as the fraction of past incidents within area a of severity s that volunteer v could have reached before the ambulance when dispatched. We compare the ambulance response time to the estimated volunteer response time to determine whether a volunteer would have been able to reach a specific incident before the ambulance. Note that volunteers can only arrive before the ambulance when they are within 10 kilometers of the incident as otherwise they would not have been dispatched.

The ambulance response time is provided for each incident. We estimate the hypothetical volunteer response time by adding the volunteer triage delay and the volunteer travel time. The volunteer triage delay, defined as the time between receiving the emergency call and dispatching the volunteer, can be calculated directly using the incident data. The volunteer travel time is estimated based on the Euclidean distance between the incident and the volunteer's base location. Although volunteers can respond from a different location than the provided base location, this is very rare and cannot be predicted up front. Hence, volunteers are assumed to always respond from the provided base locations.

We use the incidents of the nearest area to estimate the relief probabilities for areas without any incidents, where the original incident locations are replaced with the area centroids when calculating the distances.

As Lincolnshire is a very rural area, volunteers travel to incidents almost exclusively by car. Based on the findings of Auricchio et al. [1], we therefore assume that volunteers travel with an average speed of 40 km/h. Note that we always use the ambulance response time and volunteer triage delay of the same incident to control for any correlation between the two. Moreover, note that we do not incorporate an acceptance delay (i.e. the time between volunteer notification and acceptance) when estimating the hypothetical volunteer response time. This is because LIVES' CFRs are called on a dedicated phone when dispatched, causing any acceptance delay to be negligible.

F Numerical results for expected coverage optimization

When optimizing expected coverage, $p_{v,a,s,i}$ is defined as the probability that volunteer v can cover an incident of severity s occurring in area a during interval i. Here, a volunteer covers a specific incident if the volunteer arrives before a pre-defined response time target. These coverage parameters are estimated similarly to the probabilities with which volunteers arrive before the ambulance, with the only difference of using fixed response-time targets instead of the ambulance arrival times. We use the same response-time targets as currently adopted by the NHS, which are 15 minutes for severity 1 incidents, 40 minutes for severity 2 incidents, and 120 minutes for severity 3 incidents.

F.1 Optimizing a one-year budget

The expected coverage before training any volunteer is 0.190, meaning that for roughly 19% of the incidents at least one volunteer can arrive within the corresponding response-time target. As with the expected relief, the expected coverage before the training of volunteers varies considerably per area as can be seen in Figure 4a.

Optimally spending a one-year budget increases the expected coverage to 0.249, a 30.9% improvement compared to before training any volunteer. The expected coverage would increase to 0.271 when training all volunteers to level 4. This implies that over 72% of the potential increase in the expected coverage can already be obtained by spending a one-year budget. Moreover, as can be seen in Figure 4c, the increase in expected coverage also varies considerably per area.

As can be seen in Figure 4 and Table 1, the results for the expected coverage optimization are very similar to the results for the expected relief optimization. In fact, the optimal solution for the expected coverage optimization is exactly the same as the optimal solution for the expected relief optimization given a one-year budget; the same volunteers are trained and all volunteers selected to be trained are trained to level 4. Although this is not necessarily the case when a slightly different budget is used, the optimal solutions are very similar in terms of which volunteers are trained and to which training level, regardless of the specific budget used. In this case study, the insights obtained regarding volunteer selection for expected relief optimization thus also extend the expected coverage optimization.

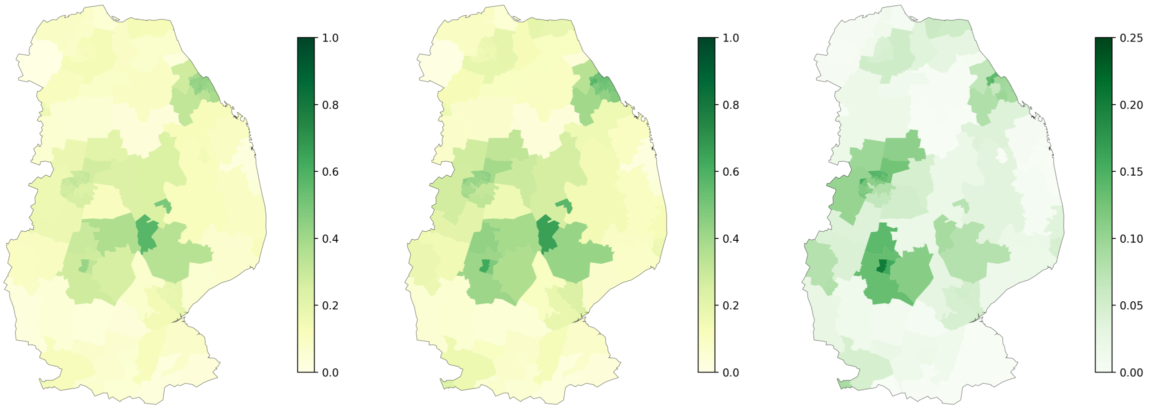

(a) Expected coverage before training

(b) Expected coverage after training

(c) Absolute increase in expected coverage

Figure 4: Results of the optimization model for a one-year budget

Table 1: Average expected coverage per required training level

	Level 2	Level 3	Level 4
Expected coverage before training	0.269	0.216	0.112
Expected coverage after training	0.271	0.252	0.230

F.2 Performance of the alternative training strategies

Besides the optimization model we also analyze three alternative training strategies. These strategies greedily select volunteers based on one specific important characteristic that impacts the benefit of training volunteers. Specifically, the availability heuristic greedily selects the volunteer with the highest average probability of being available, the demand heuristic greedily selects the volunteer with the most amount of past incidents that have occurred within 10 km, and the remoteness heuristic greedily selects the volunteer for which the incidents that have occurred within 10 km have the smallest average number of other volunteers located within 10 km. Here, we analyze the performance of these alternative strategies.

The expected coverage of the solutions obtained by the alternative training strategies and the corresponding relative improvement ratios as a function of the available budget are provided in Figure 5. As can be seen, the results displayed in Figure 5 are very similar to the results for the expected relief optimization. Hence, the performance of the alternative training strategies is very similar when optimizing the expected coverage compared to optimizing the expected relief; there is a clear order in the performance of the three strategies and the optimality gaps are of similar magnitude. As a result, the insights obtained regarding the performance of the alternative training strategies also extend from the expected relief optimization to the expected coverage optimization.

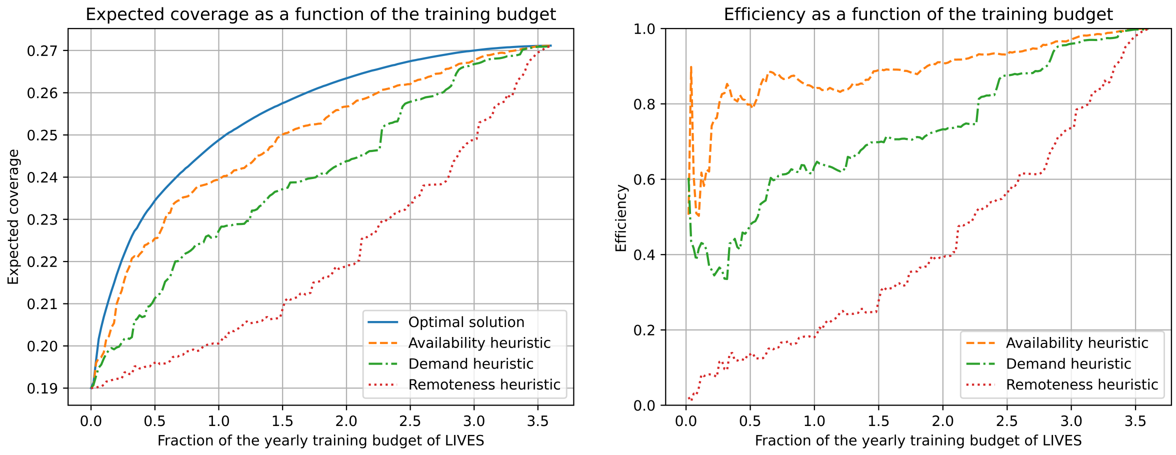

(a) Objective values

(b) Relative improvement ratios, measured as the fraction of the optimal expected coverage improvement obtained with the same budget

Figure 5: Performance of the alternative training strategies for expected coverage optimization

G Numerical results with additional dispatch restrictions

This section provides the numerical results for the expected relief optimization with additional dispatch restrictions. We specifically consider two scenarios. In the first scenario, volunteers can only be dispatched to incidents requiring up to one level lower than their own. For instance, a level 4 volunteer may be dispatched to incidents requiring level 3 or 4, but not to incidents requiring level 2. In the second scenario, volunteers can only be dispatched to incidents that correspond exactly to their training level. The optimization model can accommodate these scenarios rather easily by adjusting the constraint linking the x and z variables.

The results of the two scenarios, as well as the results for the baseline case without dispatch restrictions, are provided in Table 2. These results show that imposing additional dispatch restrictions substantially reduces expected relief, as fewer volunteers qualify for deployment to each incident. Moreover, the relative improvement obtained by optimally training volunteers decreases when the imposed restrictions become stricter.

Table 2: Optimization results when imposing additional dispatching restrictions

	Baseline	First scenario	Second scenario
Expected relief before training	0.169	0.120	0.069
Expected relief after training	0.228	0.150	0.083
Relative improvement	34.7%	25.6%	19.1%
Number of volunteers trained	38	57	39
Number of volunteers trained to level 3	0	38	0
Number of volunteers trained to level 4	38	19	39

The training strategies of the different scenarios display some interesting differences. In the first scenario, it is no longer optimal to train all volunteers to level 4. This is because volunteers of level 4 cannot be dispatched to incidents of level 2, and therefore, training volunteers to level 4 would decrease the expected relief for incidents requiring level 2. Additionally, it is possible to train more volunteers when not everybody is trained to level 4. Taken together, these factors make it more beneficial to train a larger number of volunteers, but not all to level 4, rather than training fewer volunteers all to level 4. In the second scenario, it is once again optimal to train all volunteers to level 4. This mainly reflects the fact that more incidents require level 4 than level 2 or 3.

The second main difference between the optimal training strategies across the different scenarios involves *which* volunteers are selected to be trained. The availability of volunteers remains the most important characteristic in all scenarios: in the first scenario, trained volunteers are available twice as often as untrained volunteers, while in the second scenario, trained volunteers are available almost four times as often as untrained ones. However, the scenarios differ in where trained volunteers are located. It remains optimal to train volunteers in urban areas in the first scenario. The correlation between expected relief improvement and population density is almost identical to that in the baseline case (the correlation coefficient is 0.489 in the baseline case vs. 0.468 in the first scenario). In the second scenario, however, trained volunteers are much more evenly spread across urban and rural areas; the correlation between expected relief improvement and population density is almost three times smaller compared to the other scenarios (the correlation coefficient is 0.166). This finding can mainly be explained by the fact that training volunteers to level 4 decreases the expected relief for incidents requiring level 2 or 3. This makes it suboptimal to train many volunteers in the same area to the highest level, regardless of the urbanity of the area, simply because the relief reduction for lower levels would be too large. Instead, it is optimal to create a more even distribution of volunteers across the training levels in many areas, both urban and rural.

These results thus show that although the availability of volunteers remains the most important characteristic based on which volunteers are selected to be trained, the dispatch restrictions can have important implications for how much, where, and to what level volunteers should be trained.

References

[1] Auricchio, A., Gianquintieri, L., Burkart, R., Benvenuti, C., Muschietti, S., Peluso, S., Mira, A., Moccetti, T., and Caputo, M. L. (2019). Real-life time and distance covered by lay first responders alerted by means of smartphone-application: Implications for early initiation of cardiopulmonary resuscitation and access to automatic external defibrillators. *Resuscitation*, 141:182–187.

[2] HiGHS (2024). HiGHS - high performance software for linear optimization. https://highs.dev/. Accessed October 14, 2024.

[3] Office for National Statistics (2024). Middle layer Super Output Area population estimates (supporting information). https://www.ons.gov.uk/peoplepopulationandcommunity/populationandmigration/populationestimates/datasets/middlesuperoutputareamidyearpopulationestimates. Accessed October 10, 2024.
